# Accessing Open PHACTS: interactive exploration of compounds and targets from the semantic web

**DOI:** 10.1186/1758-2946-6-S1-P52

**Published:** 2014-03-11

**Authors:** Katrin Stierand, Tim Harder, Lothar Wissler, Christian Lemmen, Matthias Rarey

**Affiliations:** 1 Center for Bioinformatics (ZBH), University of Hamburg, Bundesstr. 43, 20146 Hamburg, Germany; 2 BioSolveIT GmbH, An der Ziegelei 79, 53757 Sankt Augustin, Germany

## 

Pharmacological research is hampered by scattered data which have to be retrieved by varying methods and in different data formats. This heterogeneity increases research costs and limits throughput. Over the last two years, the Open PHACTS Discovery Platform [1] has been developed as a centralized repository, integrating pharmacological data from a variety of information resources and providing tools and services to query these integrated data in pharmacological research.

Following an application-oriented approach, the Open PHACTS project started with the definition of potential use cases in the form of prioritized research questions [2], most of which can only be answered by accessing multiple data sources in the web. The development of the platform as well as the services has been guided by these questions.

Here, we present the ChemBioNavigator (CBN) [3], a web application allowing to navigate the Open PHACTS chem-bio space with a focus on small molecules and their targets. CBN comprises of a large visualization area with different view modes and two information panels, allowing a deeper insight in information for compounds and targets. It allows interactive exploration of compound sets through sorting and subset selection as well as extending sets by substructure or similarity search. The relation between compounds and targets is defined by assay data from the Discovery Platform. Each compound and each target is annotated with information from multiple data sources which is provided together with the provenance for each data point.

In this contribution we roughly outline the OpenPHACTS/CBN technology and present a number of high-priority research questions, highlight the advantages of exploiting the integrated data through the CBN's smart and intuitive interface.
